# *In Vitro* Recapitulation of Functional Microvessels for the Study of Endothelial Shear Response, Nitric Oxide and [Ca^2+^]_i_

**DOI:** 10.1371/journal.pone.0126797

**Published:** 2015-05-12

**Authors:** Xiang Li, Sulei Xu, Pingnian He, Yuxin Liu

**Affiliations:** 1 Department of Cellular and Molecular Physiology, Penn State University, School of Medicine, Hershey, Pennsylvania, United States of America; 2 Lane Department of Computer Science and Electrical Engineering, West Virginia University, Morgantown, West Virginia, United States of America; Hungarian Academy of Sciences, HUNGARY

## Abstract

Microfluidic technologies enable *in vitro* studies to closely simulate *in vivo* microvessel environment with complexity. Such method overcomes certain constrains of the statically cultured endothelial monolayers and enables the cells grow under physiological range of shear flow with geometry similar to microvessels *in vivo*. However, there are still existing knowledge gaps and lack of convincing evidence to demonstrate and quantify key biological features of the microfluidic microvessels. In this paper, using advanced micromanufacturing and microfluidic technologies, we presented an engineered microvessel model that mimicked the dimensions and network structures of *in vivo* microvessels with a long-term and continuous perfusion capability, as well as high-resolution and real-time imaging capability. Through direct comparisons with studies conducted in intact microvessels, our results demonstrated that the cultured microvessels formed under perfused conditions recapitulated certain key features of the microvessels *in vivo*. In particular, primary human umbilical vein endothelial cells were successfully cultured the entire inner surfaces of the microchannel network with well-developed junctions indicated by VE-cadherin staining. The morphological and proliferative responses of endothelial cells to shear stresses were quantified under different flow conditions which was simulated with three-dimensional shear dependent numerical flow model. Furthermore, we successfully measured agonist-induced changes in intracellular Ca^2+^ concentration and nitric oxide production at individual endothelial cell levels using fluorescence imaging. The results were comparable to those derived from individually perfused intact venules. With *in vivo* validation of its functionalities, our microfluidic model demonstrates a great potential for biological applications and bridges the gaps between *in vitro* and *in vivo* microvascular research.

## Introduction

The development of microfluidic devices has been embraced by engineers over two decades. However, the adaptation and application of microfluidics in mainstream biology is still lacking. According to the recent summary, the majority publications of microfluidics are still in engineering journals (85%) [1]. The improved performance of microfluidic devices have not been well accepted by many biologists and applied to biological studies [1, 2]. More experimental evidence is needed to demonstrate that microfluidics has the advantage over the conventional transwell assays and macroscale culture dish/glass slide approaches for developing more physiologically relevant *in vitro* microvessel model. In this paper we continue our previous efforts in developing *in vitro* functional microvessels that could provide a platform for the study of complex vascular phenomena [3].

Several groups have pioneered in the development of advanced microvessel models using micromanufacturing and microfluidic techniques [4–8]. Each of those microvessel models demonstrated unique features and biological applications, such as the use of either polymer or hydrogel to template the growth of vascular endothelial cells (ECs) [4], co-cultured ECs with other vascular cells [5], simulating the vascular geometry pattern and studying vascular geometry associated endothelial leukocyte interactions [8], as well as investigating EC involved angiogenesis and thrombosis [5–7]. However, there have been very limited reports for microvascular function related changes in endothelial cell signaling in microfluidic based systems.

Nitric oxide (NO) is essential for controlling vascular tone and resistance in arterioles, and regulating vascular wall adhesiveness and permeability in venules [9–12]. Additionally, the endothelial intracellular Ca^2+^ concentration [Ca^2+^]_i_ has been recognized to play an important role in microvessel permeability [11, 13–18], angiogenesis [19] and morphogenesis [20]. Although a few studies previously reported the use of DAF-2 DA in microfluidic network, some of them only showed DAF-2 loading [21, 22], and others were lack of appropriate resolution and data analysis [23]. Up-to-date, the agonist-induced dynamic changes in endothelial [Ca^2+^]_i_ and NO production have not been well demonstrated in previous microfluidic based studies, especially no quantitative measurements were conducted with temporal and spatial resolution.

In this paper, we presented an *in vitro* formation of a microvessel network and directly compared the key features with the results derived from microvessels *in vivo*. Continuous microfluidic perfusion is able to control the mass transfer and flow shear stresses precisely. A confluent endothelial monolayer was formed and fully covered inside the entire microchannel network. The vascular endothelial adherens junctions were confirmed by VE-cadherin immunofluorescence staining. Cell morphology changes in response to different patterns of shear stress were evaluated by staining endothelial cell F-actin. Additionally, following our established methods developed in individually perfused microvessel, endothelial [Ca^2+^]_i_ and NO production were quantitatively measured using a real-time and high-resolution imaging under controlled and stimulated conditions. The main objectives of this study are to develop an *in vitro* functional microvessel network, validate some of the key biological features of microvessel endothelial cells, and provide a validated *in vitro* tool for the future studies of human endothelial cells under physiological and pathological conditions.

## Materials and Methods

### Design and fabrication

**Fig 1:**
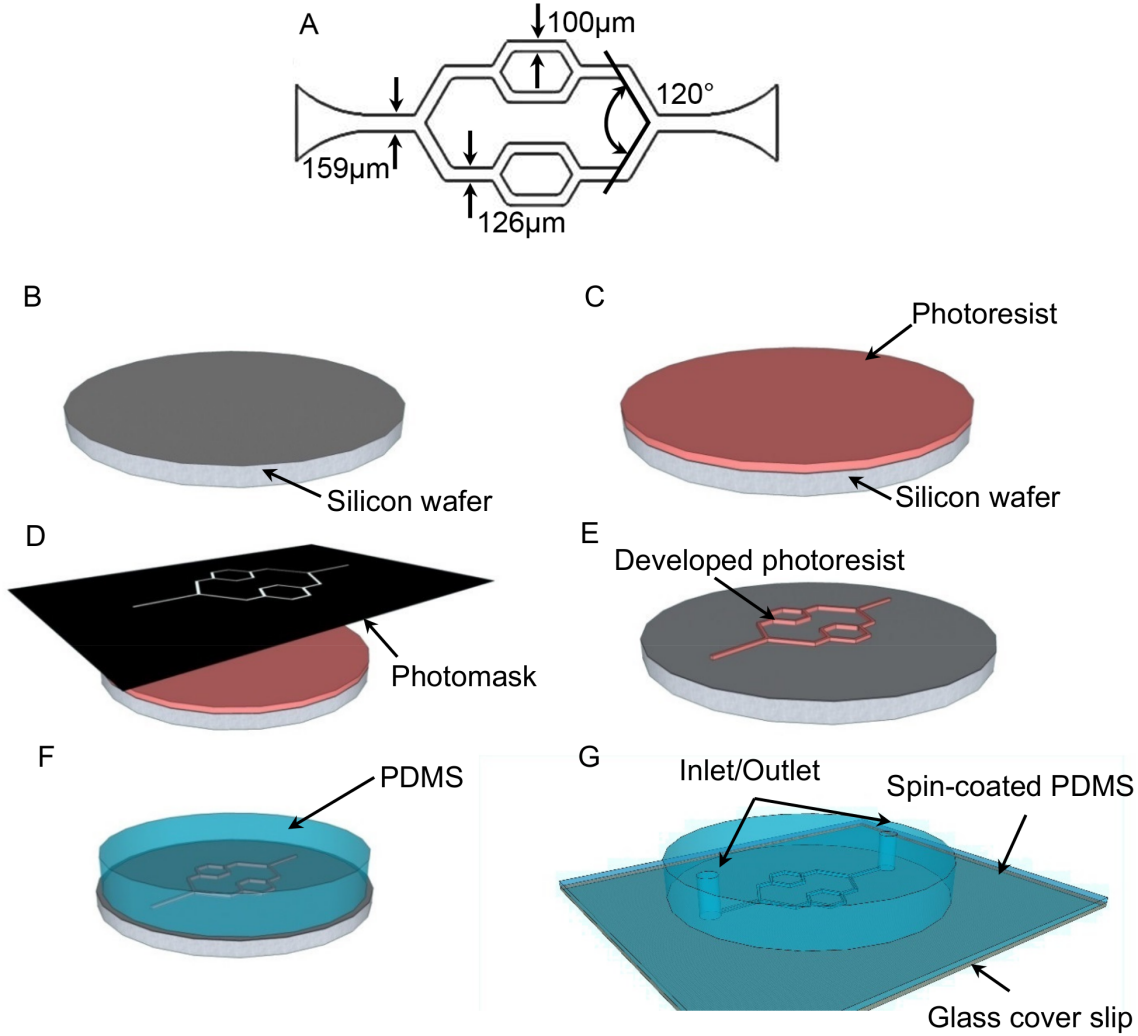
The schematic design and fabrication procedures for the microfluidic microchannel network. A. The schematic design shows the bifurcation angle and the widths of microchannels at differnet levels. B. A pre-cleaned silicon substrate. C. SU-8 photoresist was spun-coated onto the silicon wafer. D. The photoresist was exposed to UV light through the photomask. E. The developed microchannel network pattern was used as the master mold. F. PDMS mixing solution was cast onto the master mold and cured. G. The inlet and the outlet were punched and the microchannel device was bonded onto a glass substrate with a spun-coated thin PDMS layer.

The microchannel network designed in this paper was a three-level branching microchannels. As shown in Fig 1A, the width of microchannels was 100 μm, 126 μm, and 159 μm, respectively. The angles at the bifurcations was 120°. Standard photolithography was used for the master mold fabrication and polydimethylsiloxane (PDMS) soft lithography was used for the microfluidic microchannel network fabrication as shown in Fig 1B–1G [24]. Briefly, a silicon wafer was rinsed with acetone and methanol and baked on a hot plate (150°C) over 30 minutes for dehydration (Fig 1B). SU-8 photoresist (SU8-2050, Microchem, Westborough, MA USA) was spun-coated over the pre-cleaned silicon wafer with a thickness of 100 μm, and then the wafer was baked on the hot plate at 65°C and 95°C, respectively (Fig 1C). The designed patterns were transferred from a film mask to a SU-8 thin film after the UV light exposure (OAI model 150, San Antonio, TX USA) (Fig 1D), post baking, and the development as shown in Fig 1E. After the hard baking at 150°C, the developed patterns as the master mold were ready for PDMS soft lithography. PDMS (Slygard 184, Dow Chemical, Midland, MI USA) was mixed at a weight ratio of 10:1, and cast onto the master mold to replicate the microchannel patterns (Fig 1F). PDMS was cured and peeled off from the master mold after it was baked in an oven at 60°C for 3 hours. The inlet and the outlet, which were used for the cell loading, tubing connections, media and reagent perfusion, and waste collection, were punched with a puncher (1 mm, Miltex, Plainsboro, NJ USA). In a typical confocal microscopy system, an objective lens with high numerical apertures (NA) has a limited working distance in a range of a few hundred microns [25]. Therefore, to incorporate the microfluidic devices to our confocal system, the number 1 glass coverslip (thickness of 130–160 μm, Fisher Scientific) spun-coated with a thin layer of PDMS (thickness of 20 μm) was used as the substrate for the device bonding (Fig 1G). A permanent bonding was created to seal the microchannels completely after oxygen plasma treatment (50 W, 100 mtorr) of PDMS for 30 seconds.

### Viscosity measurement and numerical simulation

**Fig 2:**
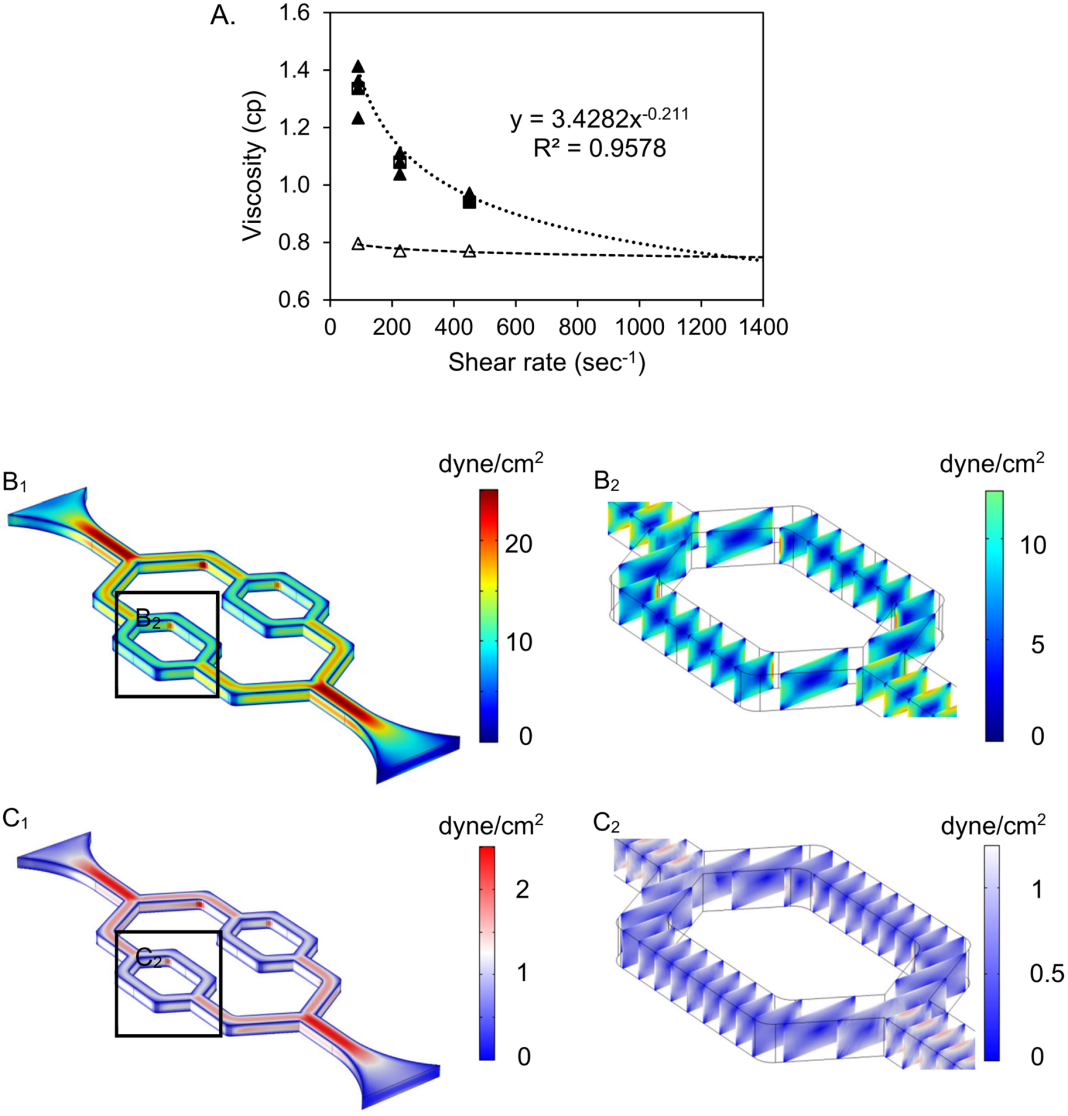
Viscosity measurement of culture media and numerical simulation for the wall shear stress distribution of the microchannel network. A. Viscosity measurement of culture media perfusate. Filled triangles (▲) represent viscosity of the culture medium with 10% FBS; Open triangles (△) represent the viscosity of standard Newtonian calibration solution. Dotted and dashed lines are their trend lines, respectively. B_1_. COMSOL simulation shows the wall shear stress distribution through the entire network under high flow rate condition. B_2_. The wall shear stress distribution of the selected region in B_1_. C_1_. COMSOL simulation shows the wall shear stress distribution through the entire network under low flow rate condition. C_2_. The wall shear stress distribution of the selected region in C_1_.

To estimate the shear stresses of the culture media under the experimental conditions, the viscosity of the cell culture medium containing 10% fetal bovine serum (FBS) was measured using a Wells-Brookfield cone/plate digital viscometer (LVTDCP, cone # CP-40, Stoughton, MA, USA) at 37°C, with shear rate at 90, 225, 450 sec^-1^, respectively. As shown in Fig 2A, the media viscosity (5 repeated measurements) measured at the shear rate range showed some shear rate dependence, an indication of non-Newtonian behavior. Based on these measurements, the dynamic viscosity (μ) as a function of shear rate (γ) was fitted by an equation μ = mγ^n-1^, where an exponent n is 0.789 and a flow consistency index (m) is 3.4282.

We then conducted numerical simulations using a three-dimensional, finite element model in the commercial software COMSOL Multiphysics (Version 4.0.0.982, COMSOL Inc., Burlington, MA, USA). Stationary incompressible Navier-Stokes equations were chosen as governing equations for the fluids, and no-slip wall boundary condition was set along the internal surfaces of the microchannels. The implemented boundary conditions at the inlet and outlet were constant inlet velocity and zero external pressure, respectively. Culture medium was selected as the reference fluid during the simulation with a constant density (1020 kg/m^3^), and a power law dynamic viscosity model was applied based on the fitted equation derived from measured culture media viscosities (Fig 2A). PARallel sparse DIrect linear SOlver (PARDISO) was used to execute the iteration, and the model was considered converging when the estimated error was less than 2.2e^-11^. The simulated wall shear stress distribution along the entire network and the selected regions of channels were shown in Fig 2B and 2C, respectively.

### Cell culture

Primary human umbilical vein endothelial cells (HUVECs) were purchased from Lonza. The cells were maintained in MCDB 131 culture medium (Gibco, Carlsbad, CA USA) supplemented with 10% FBS, 1% L-glutamine, 0.1% Gentamicin, 0.05% bovine brain extract (9mg/mL), 0.25% endothelial cell growth supplement (3mg/mL), and 0.1% heparin (25mg/mL) in tissue cultured flasks, which were pre-coated with 0.2% gelatin. The cell culture was performed in a humidified atmosphere of 5% CO_2_ at 37°C, and the cells between passage 2 and passage 5 were used for this study. When the cultured HUVECs reached confluent, the cells were harvested and re-suspended in 8% Dextran (mol wt 70,000, Sigma, St. Louis, MO, USA) diluted with MCDB 131 culture medium. Dextran was used to increase the medium viscosity for better controlling cell seeding inside the microchannels.

Prior to cell loading, the device was treated with oxygen plasma for 3–5 minutes to reduce the hydrophobicity of inner surfaces of PDMS microchannels. The device was then loaded with deionized water and sterilized under the UV light exposure for 8 hours in a laminar biosafety hood. After UV sterilization, the device was rinsed with 1× phosphate buffered saline (PBS), coated with fibronectin diluted in 1×PBS (100 μg/mL, Gibco, Carlsbad, CA USA) along the entire inner surfaces of PDMS channel walls, and incubated at 4°C inside a refrigerator for overnight. After this, the device was rinsed with 1×PBS again to remove the free fibronectin solution completely, and loaded with cell media. Finally, the device was incubated for 15 minutes at 37°C and was ready for cell loading.

**Fig 3:**
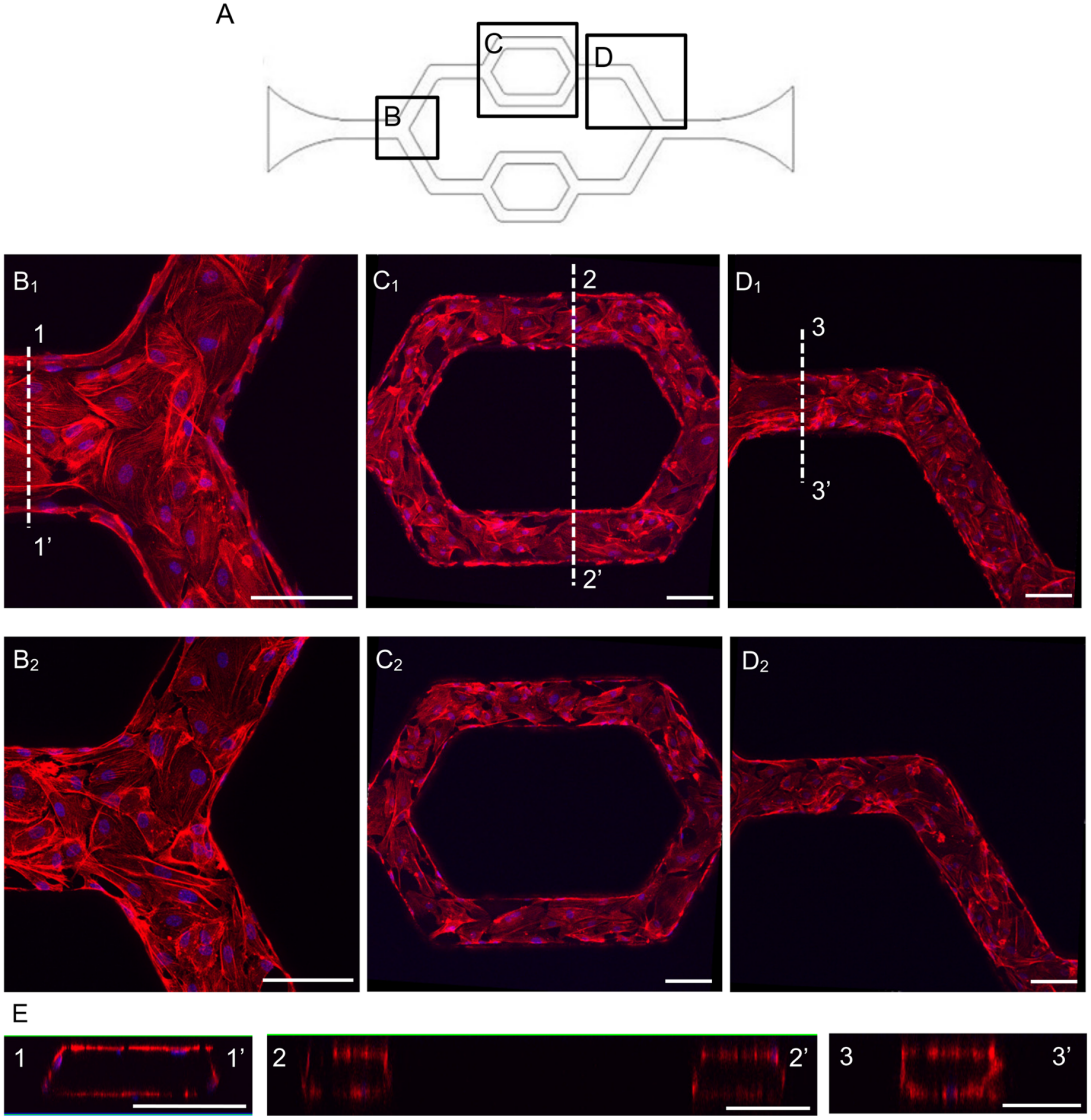
The representative confocal images show the HUVECs successfully cultured throughout the inner surfaces of the entire microchannel network. A. The schematic image of the network with selected regions as shown in B-D. B-D. HUVECs stained with F-actin and cell nuclei in each region, where B_1_, C_1_ and D_1_ show the upper layer of endothelial cells at different location of the channel. B_2_, C_2_ and D_2_ show the lower layer of the endothelial cells at different locations of the channel. E. The three-dimensional reconstructed cross-sectional images at each region. The locations of the cross-sections are indicated as 1–1’, 2–2’, and 3–3’ in B_1_-D_1_, respectively. Each scale bar is 100 μm.

To load the cells, a droplet (10 μL) of HUVECs was placed at the inlet, and a slow flow was created by either tilting the device, or placing a glass pasteur pipette (the inner diameter of the pipets is around 1.5 mm, VWR) at the outlet. Capillary action through the microchannels was gently introduced by the glass pipette and the cells slowly moved along the media into the channels. The key for a successful cell loading was to control the flow velocity very slowly, otherwise, most of the cells cannot attach uniformly inside the microchannels. After 15–20 minutes incubation in the incubator, the attached cells on the PDMS channel walls can be visually confirmed under the microscope. An additional loading can be performed if necessary. After a satisfied cell seeding density was reached, the device was gently rinsed with the media to remove the dextran solution. A complete attachment requires five to six hours. Long-term continuous perfusion was set up by a syringe pump system (Harvard Apparatus, Holliston, MA, USA) with a steady flow rate of 0.35 μL/min. The perfusion can last up to two weeks, and can be adjusted to maintain different flow patterns if necessary [3]. The attached cells were grown under the perfusion along the entire inner surfaces of microchannels, which was illustrated by F-actin staining. Fig 3 shows the confocal images of endothelial cell F-actin at upper and lower layers of endothelial cells within the channels, as well as the three-dimensional reconstructed cross sectional images at different channel regions. The studies of cell morphology, endothelial cell junction, endothelial [Ca^2+^]_i_, and NO responses to agonist were conducted on either the lower or upper surfaces of the microchannels and no significant differences were observed between upper and lower stack of images.

### Confocal fluorescence imaging of intracellular calcium concentration ([Ca^2+^]_i_)

Endothelial [Ca^2+^]_i_ was measured in Fluo-4 AM loaded endothelial cells on a Leica TCS SL confocal microscope with a Leica ×25 objective (NA: 0.95). An argon laser (488 nm) at 50% power was used for excitation, and the emission band was 510–530 nm. To minimize photobleaching, fluo-4 images were collected using a 512 × 512 scan format at a z-step of 2 μm. Stacks of images were collected from the same group of HUVECs with 20 seconds intervals. Each network device was first loaded with fluo-4 AM (5 μM) for 40 minutes followed by albumin-Ringer perfusion to rinse the lumen fluo-4 AM before control images were collected. Quantitative analysis of endothelial [Ca^2+^]_i_ at the individual endothelial cell level was conducted using manually selected regions of interests (ROIs) along the microchannels. Each ROI covered the area of one individual endothelial cell, as indicated by the fluorescence outline. The changes in endothelial [Ca^2+^]_i_ at the cellular levels were quantified by calculating the mean fluorescence intensity (FI) of each stack of ROIs after the subtraction of the background auto fluorescence. The percent change in FI was expressed as FI/FI_0_*100, where FI_0_ was the initial baseline FI of fluo-4. Details have been described previously [11].

### Confocal fluorescence imaging of nitric oxide production

Endothelial NO levels were investigated at the cellular levels in the microvessel network using DAF-2 DA, a membrane-permeable fluorescent indicator for NO, and fluorescence imaging. Experiments were performed on a Nikon Diaphod 300 microscope equipped with a 12-bit digital CCD camera (ORCA; Hamamatsu) and a computer controlled shutter (Lambda 10–2; Sutter Instrument; Novato, CA). A 75-W xenon lamp was used as the light source. The excitation wavelength for DAF-2 was selected by an interference filter (480/40 nm), and emission was separated by a dichroic mirror (505 nm) and a band-pass barrier (535/50 nM). All the images were acquired and analysed using Metafluor software (Universal Imaging).

Each network device was first perfused with albumin-Ringer solution containing DAF-2 DA (5 μM) for 35~40 minutes before collecting DAF-2 images. DAF-2 DA was present in the perfusate throughout the experimental duration [26]. All images were collected from a group of HUVECs located in the same focal plane using a Nikon Fluor lens (x20, NA: 0.75). Data analysis was conducted at the individual endothelial cell level using manually selected ROIs. Each ROI covered the area of one individual cell as indicated by the fluorescence outline. The PDMS auto fluorescence was subtracted from all of the measured fluorescence intensities (FIs). The basal NO production rate was calculated from the slope of the mean FI increase during albumin-Ringer perfusion after DAF-2 loading was reached the steady state. The changes in FI_DAF_ upon adenosine triphosphate (ATP) stimulation were expressed as the net changes in FI (ΔFI). FI was expressed in arbitrary units (AU) and identical instrumental settings were used for all of the experiments. The rate of FI_DAF_ change was derived by first differential conversion of cumulative FI_DAF_ over time. Details have been described previously [12, 26].

### Immunofluorescent staining

HUVECs were fixed in 2% paraformaldehyde solution (Electron Microscopy Science, Hatfield, PA, USA) for 30 minutes at 4°C by perfusing the fixing solution into the network. The cells were blocked with 1 mg/mL bovine serum albumin (BSA, Sigma, St. Louis, MO, USA) in PBS solution for 30 minutes followed by permeabilization with 0.1% Triton X-100 (Sigma, St. Louis, MO, USA) for 5 minutes. The primary antibody (VE-cadherin) was perfused at 4°C for overnight. Then, the second antibody (Alexa488, Invitrogen, Carlsbad, CA, USA) was perfused for 1 hour at the room temperature. DRAQ 5 (Biostatus, Shepshed Leicestershire, UK) was used for cell nuclei staining. Following the similar fixing, blocking, and permeabilizing procedures, F-actin was labeled by perfusing phalloidin-Alexa 633 (Sigma, St. Louis, MO, USA) for 10 minutes, followed by the DRAQ5 nuclei staining. Fluorescent images were obtained using Nikon Ti-E inverted microscope (Chiyoda, Tokyo, Japan) and a confocal laser-scanning microscope (Leica TCS SL). The objective lens used for Nikon Ti-E was Nikon Plan Fluor X10, NA 0.3 Ph1 DLL. The images were acquired at 1390 × 1040 pixel and the pixel size is 0.645 μm. The objective lens used for the confocal microscope (Leica TCS SL) was Leica APO X25, NA 0.95 W CORR using 1024 × 1024 format and the pixel size is 0.58 μm.

VE-cadherin staining in individual venules was performed following single vessel perfusion procedure in the mesentery of Sprague-Dawley rats (2–3 mo old, 220 to 250 g; Hilltop Laboratory Animal, Scottdale, PA). Details have been described previously [14, 27]. In brief, the rat was anesthetized with Pentobarbital sodium, given subcutaneously with initial dosage at 65 mg/kg body wt and an additional 3 mg/dose given as needed. A midline surgical incision (1.5 to 2 cm) was made in the abdominal wall and the mesentery was gently moved out of the abdominal cavity and spread over a coverslip for single vessel perfusion. The selected venule was then cannulated and perfused with BSA-Ringer perfusate first to remove the blood in the vessel lumen before fixation. The fixation and antibody staining procedures are identical to those described in cultured microvessels.

### Cell morphology analysis in response to shear stress

To study the actin cytoskeleton and HUVECs morphology changes under shear stresses, different scenarios were performed to vary the culture and shear flow conditions. Detailed experimental conditions are listed in Table 1. Briefly, after initial seeding three different flow conditions were set for the same patterned networks in different devices as shown in Table 1: Low shear culture, low shear test (LSC-LST); Low shear culture (till the ECs reached confluence), high shear test (LSC-HST); and high shear culture, high shear test (HSC-HST). The transition from low shear stress to high shear stress was gradually applied by programming a step function (10 steps of increase in 18 hours) using the syringe pump (Harvard Apparatus, Holliston, MA, USA).

**Table 1:** Summary of flow conditions applied to cultured microvessels.

	Culture and test condition	Flow rate for culture/test (μL/min)	Wall shear stress at the selected region (dyne/cm^2^)
**LSC-LST**	Low shear culture-Low shear test	0.35/0.35	1.0
**LSC-HST**	Low shear culture-High shear test	0.35/4.05	1.0, then 10
**HSC-HST**	High shear culture-High shear test	4.05/4.05	10

Quantitative analysis of F-actin staining images was performed to examine HUVEC morphology changes (i.e. cell surface area) in responses to different levels of shear stresses. The surface area for each individual cell was acquired by performing area measurement function using NIS Elements software (Nikon, Chiyoda, Tokyo, Japan) with manually adjusting of ROIs. Each ROI covered the area of one individual cell. For statistical analysis, data was presented as the mean ± standard error (SE) and each individual experiment was performed at least three times (n ≥ 3). The results were evaluated by the t test and single factor analysis of variance (ANOVA).

## Results

### Characterization of endothelial adherens junctions in microvessels developed in microchannel network

**Fig 4:**
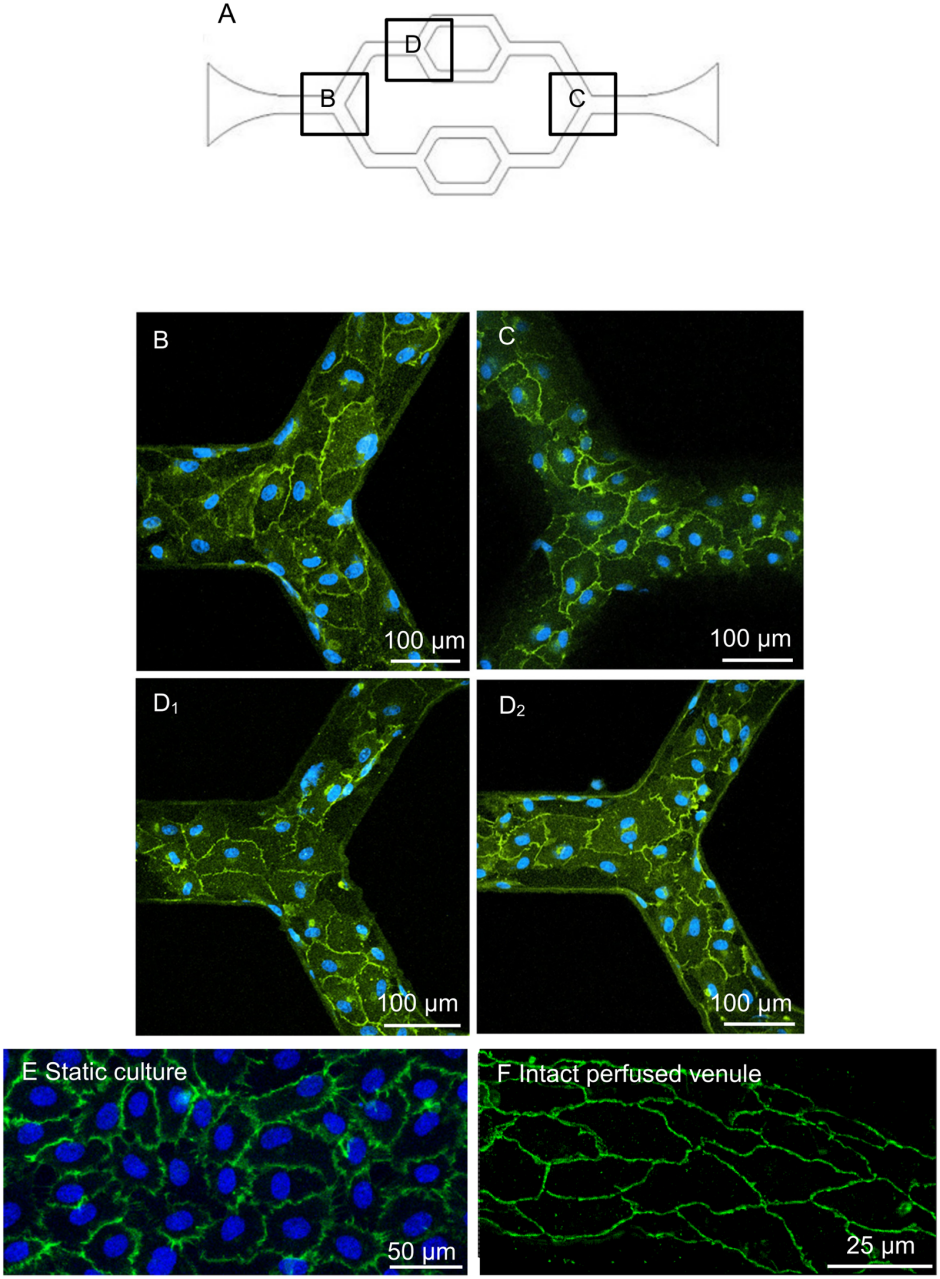
The representative immunofluorescent staining confocal images demonstrate the VE-Cadherin distributions of cultured vascular network, a statically cultured endothelial monolayer, and an intact rat mesenteric venule. A. The schematic image of the network with selected regions as shown in B-D. B-C. VE-Cadherin and cell nuclei staining were shown at the first and third branching regions. D_1_-D_2_. VE-Cadherin and cell nuclei staining were shown at the top and bottom surfaces of the second branching regions, respectively. E. VE-cadherin junction distribution of HUVECs cultured under static condition. F. VE-cadherin distribution of endothelial cells from an individually perfused intact rat venule.

With the initial cell loading concentrations of 2 ~ 4 × 10^6^ cells/mL, confluent monolayers developed within 3–4 days under a constant flow of culture media. The three-dimensional images of F-actin staining are shown in Fig 3. Under the same culture conditions, we examined the junctional formation between ECs as an indication of endothelial barrier function. VE-cadherin, an important adhesion protein for the maintenance and control of the junctions between endothelial cells, was illustrated with antibody staining. To make a direct comparison of VE-cadherin distribution between cultured ECs grown under static and continuous flow conditions, and EC junctions in intact microvessels, we also conducted VE-cadherin staining in statically cultured HUVECs and in intact venules of rat mesentery. Fig 4B–4D shows the confocal images of VE-cadherin and nuclei staining at different regions of the microchannel. The confocal images illustrate that VE-cadherin was well developed throughout the entire network, demonstrating a continuous distribution between ECs with less lattice-like structure as that often appeared in statically cultured endothelial monolayers (Fig 4E). The smooth and continuous VE-cadherin pattern shown in the microfluidic microvessels is similar to that observed in intact microvessels (Fig 4F), suggesting that the continuous flow condition during cell growth provide a better environment for appropriate EC spreading, viability, proliferation, and formation of junctions.

### Endothelial cell responses to shear stress

**Fig 5:**
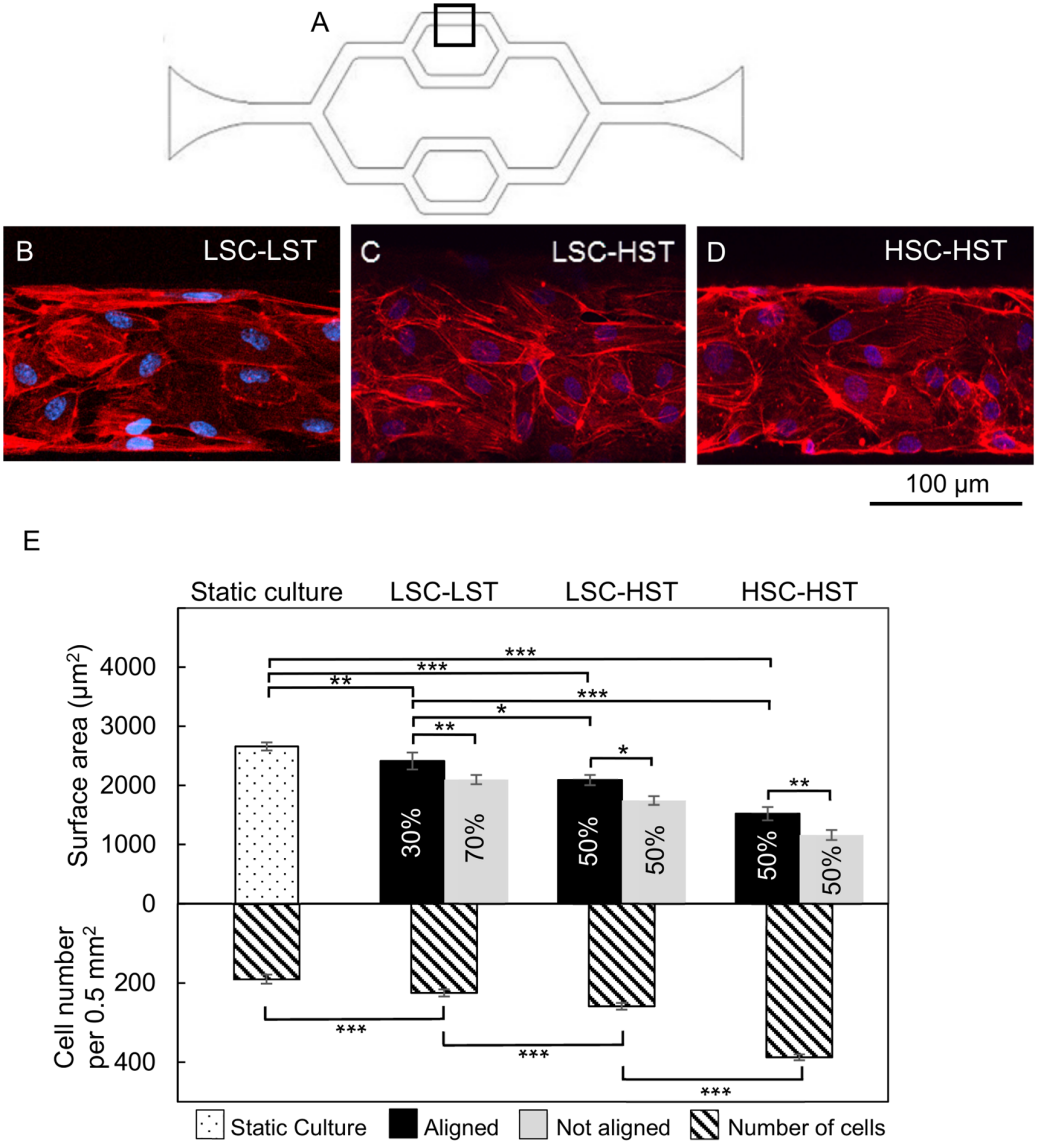
HUVECs’ responses to different levels of shear stress: The morphology changes illustrated by F-actin staining. A. The schematic image of the network showing the selected region for shear stress test. B-D. Confocal images show F-actin redistribution under different perfusion conditions. E. Comparison of the cell surface areas (upper Y axis) and cell density (number of cells per 0.5 mm^2^, lower Y axis) between static culture and the three testing groups: LSC-LST (419 cells in 6 devices); LSC-HST (412 cells in 6 devices); and HSC-HST (235 cells in 3 devices). The percentage of aligned and non-aligned cells are noted on each of the bar. All data are reported as the means ± SE of independent experiment. *: P<0.05; **: P<0.01; ***: P<0.001.

Flow related shear stress has been shown to induce redistribution of F-actin in aortic vessel segment [28], and changes in cell shape and cytoskeletal structure in cultured ECs [29]. To quantify the shear stresses within the cultured microvessel network, the numerical simulation was conducted as those shown in Fig 2B and 2C. The shear rate dependent non-Newtonian culture medium simulation showed slightly larger variations of wall shear stress at the bifurcation and turning region of the microchannel networks comparing to the Newtonian flow. However, the wall shear stress within the selected straight regions in the devices (Fig 5A) were still uniformly distributed and the magnitude of wall shear stress were 10 dyne/cm^2^ under the flow rate 4.05 μL/min, and 1.0 dyne/cm^2^ under the flow rate 0.35 μL/min within the selected regions in the devices as shown in Fig 2B_2_ and 2C_2_, which are in the range of the shear stress distribution of venules *in vivo* [30, 31]. Based on these simulations, we evaluated the cytoskeletal rearrangement of F-actin fibers and cell shape changes in response to three patterns of flow related shear stress within the microchannel networks: continuous low shear without a change (LSC-LST); low shear culture with high shear exposure (LSC-HST); and continuous high shear exposure (HSC-HST). The flow rate and correlated shear stress under each condition are listed in Table 1. Under the LSC-LST conditions, about 70% of the cells showed cobblestone pattern with dominated peripheral F-actin, and 30% of the HUVECs showed elongated cell shape with increased central stress fibers aligned along the flow direction. Under LSC-HST and HSC-HST conditions, about 50% of the cells were elongated with distinct stress fibers along the flow direction. Fig 5B–5D shows representative images from each group and Fig 5E shows the quantifications of their changes in cell surface areas in those aligned and non-aligned cells and cell density. Both aligned and non-aligned cells in three groups demonstrated shear magnitude-dependent reduction of cell surface area and corresponding increases in cell density, suggesting a role of shear stress in promoting cell proliferation.

### Measurements of endothelial calcium concentration ([Ca^2+^]_i_) in response to ATP

**Fig 6:**
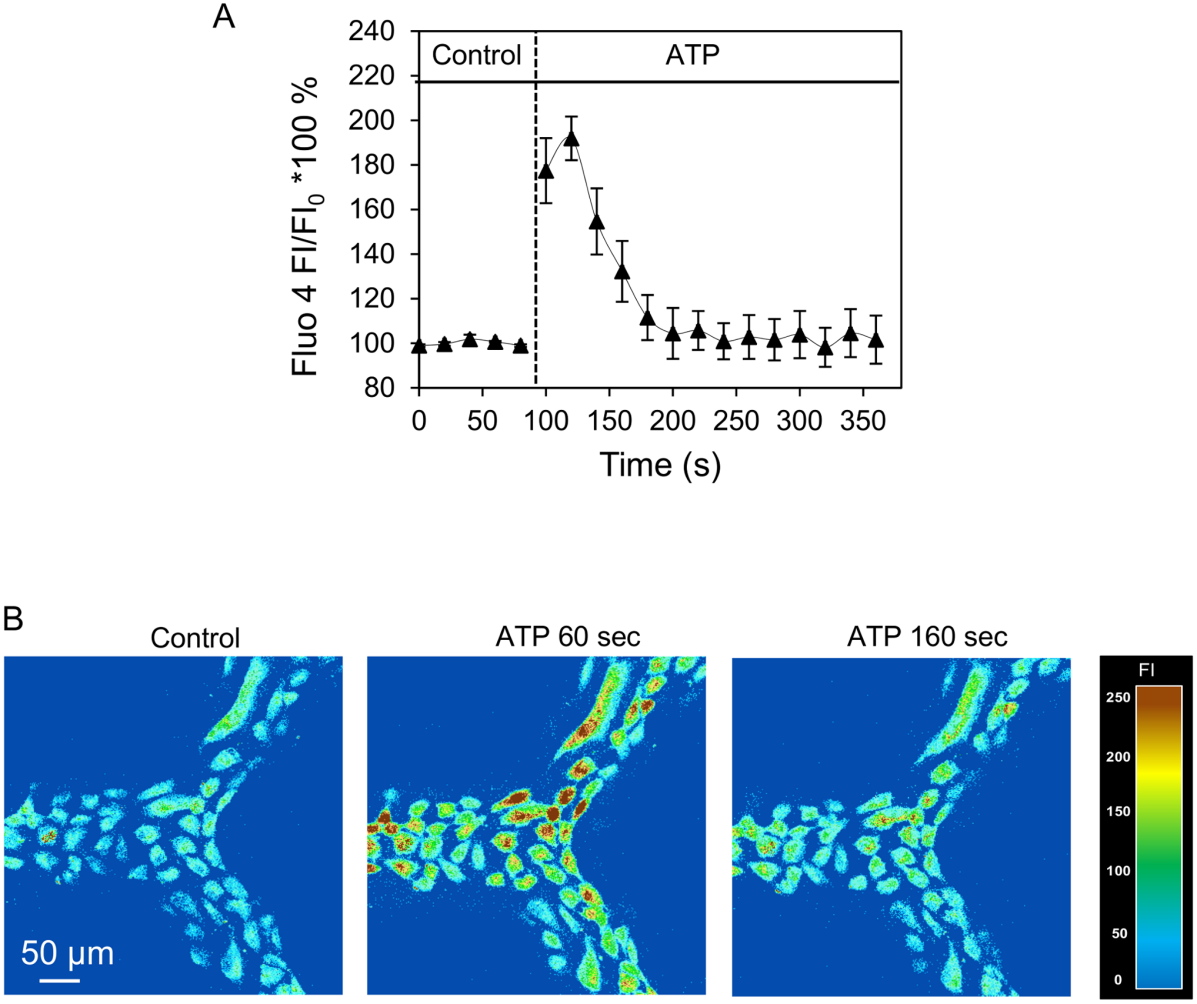
ATP-induced endothelial cell [Ca^2+^]_i_ increase in HUVECs cultured microchannel network. A. Time course of ATP-induced changes in endothelial [Ca^2+^]_i_ from a representative experiment. B. Fluo-4 confocal images from one representative experiment before and after the start of ATP perfusion at 60 seconds and 160 seconds.

Increases in endothelial [Ca^2+^]_i_ have been demonstrated to play important roles in regulation of a variety of microvessel functions including endothelial barrier function, i.e. microvessel permeability. In individually perfused microvessels, inflammatory mediator commonly induces transient increases in endothelial [Ca^2+^]_i_ followed by transient increases in microvessel permeability. To validate the biological functions of the microvessels developed under flow in our model, we applied the method developed in individually perfused intact microvessels [11, 17] to the microvessels developed in the microchannel network and quantitatively measured the changes in endothelial [Ca^2+^]_i_ when the microvessel was exposed to ATP. Experiments were conducted in four devices with fluo-4 loaded endothelial cells. When ATP (10 μM) was perfused to each device, [Ca^2+^]_i_ in all endothelial cells under the view reached a peak at 35 ± 10 seconds with the mean peak [Ca^2+^]_i_ at 187 ± 22% of the control. Representative fluo-4 images and the fluorescence quantification are shown in Fig 6. The transient pattern is similar to what we observed in individually perfused intact microvessels [17, 18].

### Measurement of ATP-induced nitric oxide production

In intact venules, it has been demonstrated that inflammatory mediator-induced increases in endothelial [Ca^2+^]_i_ was associated with increased NO production, and both increased endothelial [Ca^2+^]_i_ and NO production contribute to the increases in microvessel permeability [11, 12]. With demonstrated ATP-induced increases in endothelial [Ca^2+^]_i_, we further measured NO production in response to ATP in DAF-2 loaded endothelial cells lining the microchannels.

**Fig 7:**
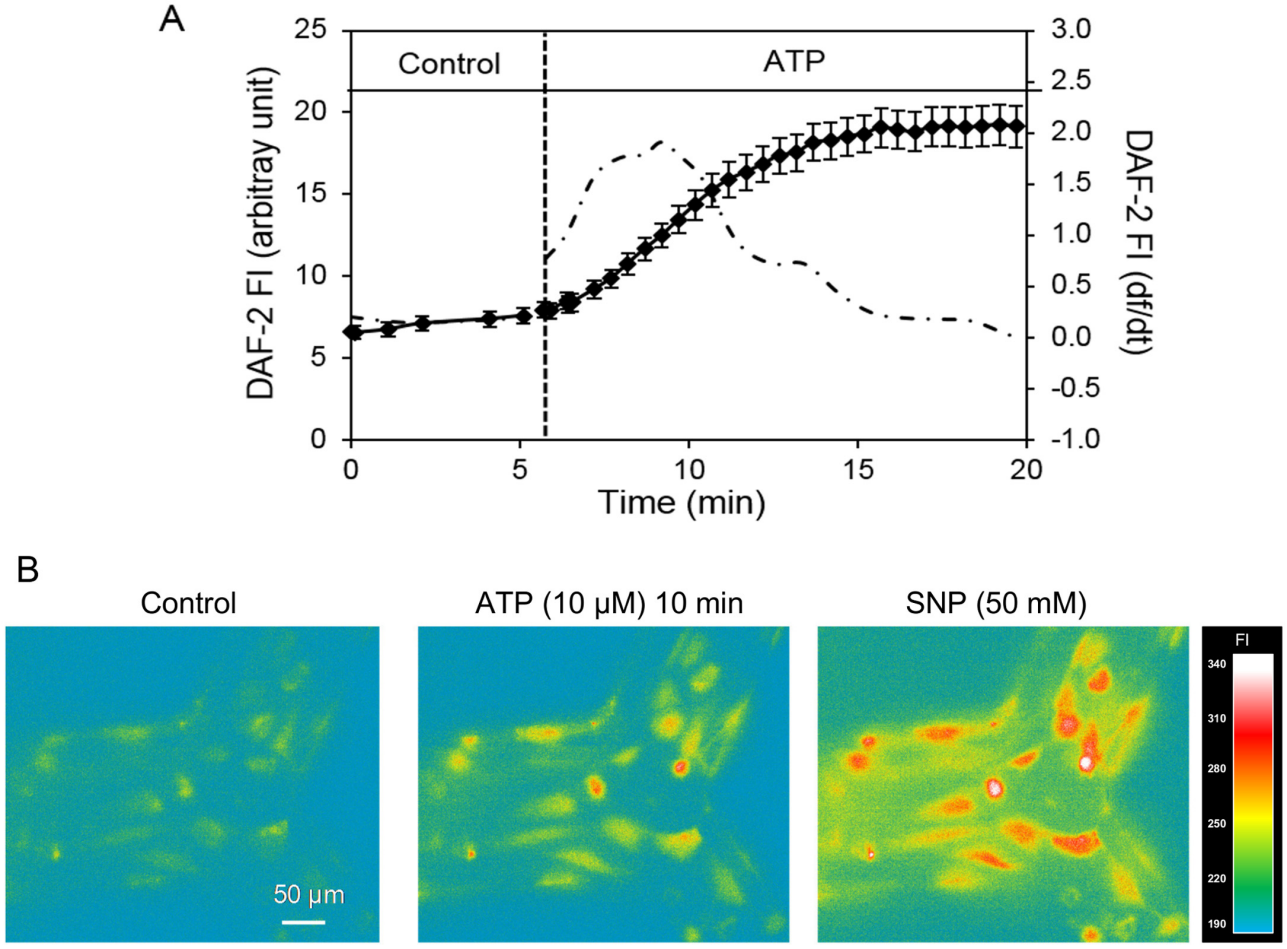
ATP-induced NO production in HUVECs cultured microchannel network. A. Time dependent changes in FI_DAF_ (left Y axis) from a representative experiment before and after ATP (10 μM) stimulation. The 1st differential conversion of cumulative FI_DAF_ (df /dt, right Y axis) represents the NO production rate (dash-dotted line). B. Representative DAF-2 fluorescence images. ATP induced significant increases in nitric oxide (NO)-DAF-2 fluorescence intensity (FI) relative to control (left). FI_DAF_ was further increased with the application of the NO donor sodium nitroprusside (SNP).

Each device was first perfused with albumin-Ringer solution containing DAF-2 DA (5 μM) for 35~40 minutes before collecting images. After the loading phase, the rate change of DAF-2 fluorescence (FI_DAF_) under control conditions was 0.15 ± 0.05 AU per min. When ATP (10 μM) was added to the perfusate, the rate change in FI_DAF_ was significantly increased. The peak rate of FI_DAF_ increase was 1.18 ± 0.37 AU per min. The increased FI_DAF_ returned to the control level after 10~15 minutes of ATP exposure. Data were derived from 3 devices with a total of 35 ROIs and 11 to 12 ROIs per vessel. NO donor, sodium nitroprusside (SNP), was added at the end of each experiment to verify the sufficiency of DAF-2 in the cells and the specificity of DAF-2 in response to NO. Fig 7 shows the quantification of time-dependent changes in FI_DAF_ and correlated images from an individual experiment. The changes in slopes of the FI_DAF_ indicate the changes in NO production rates before and after ATP stimulation. The rate of FI_DAF_ increase measured under control conditions is close to those observed in intact microvessels [9, 26, 32], indicating similar levels of basal NO production. Images in Fig 7B illustrate the ATP-induced cumulated increases in FI_DAF_ at 10 minutes of ATP exposure.

## Discussion

Our study presented *in vitro* formation of a microvessel network using a microfluidic device and characterized endothelial junctions, cellular responses to shear stresses, and quantitatively measured the changes in endothelial [Ca^2+^]_i_ and NO production before and after exposure to ATP. The results demonstrated that this engineered microvessel network recapitulates the key aspects and fundamental functions of *in vivo* venules, and reveals its promising utility for biological applications including studies with human samples.

With advanced microfabrication techniques, the flexibility to create patterns and scale sizes provides users with arbitrary design options to mimic the geometries of *in vivo* microvasculature, which allows the functional studies involved in complex flow patterns found at vessel bifurcations and in regions of high curvature that occur *in vivo*. This microfluidic microvessel network on a thin glass substrate with a spin-coated PDMS thin film (average total thickness is 150–180 μm) allows excellent light transmission and easy adaptation to both bright field and fluorescence microscopy when using short working distance and large numerical aperture objectives. Our study demonstrated that this device is capable for real-time and high-resolution imaging to detect changes in intracellular molecules and cell morphology using fluorescent markers, which is a necessary feature for biological studies. This modification is a further progress of applying our previous studies using endothelialized microvessel network [3, 24].

The main difference of using this microchannel network compared with traditional cell culture is that the cells, instead of growing under static conditions at 2-dimensional flat surface, were growing in a three-dimensional channel with continuous flow, which made the cell culture environment closer to the *in vivo* situation. By conducting direct comparisons of cell culture under static conditions and under flow in a microchannel with intact microvessels, the cultured microvessels showed junctional formation and cellular responses closer to *in vivo* than traditional static EC monolayers. Our viscosity measurements showed non-Newtonian behavior of cultured media under the physiological shear range in venules. The numerical simulation, with the implemented dynamic viscosity of the culture media, provided more accurate estimation of the wall shear stress distribution in the cultured microvessels. Based on the channel dimensions and simulation, the applied shear stress was easily controlled within the range of venous system (1 to 10 dyne/cm^2^) [30, 31]. More importantly, our study characterized the biological functions of these cultured microvessels under basal and stimulated conditions, and validated its utility for biological applications. Our results indicated that the endothelial cells grown under flow conditions formed better monolayers with well-developed junctions between endothelial cells as that demonstrated by VE-cadherin staining. As shown in Fig 3, the HUVECs completely covered the entire inner surfaces of the channels including the corners and formed a completely enclosed network. Most importantly, the VE-cadherin staining showed a uniformly distributed band between endothelial cells within the network, which is similar to that observed in intact venules (Fig 4F) [14, 27], and distinct from the intricate lattice-like structure commonly found in statically cultured endothelial monolayers (Fig 4E).

Previous studies of the endothelial F-actin rearrangement under shear stress are commonly performed in flow chambers [33, 34] and cone-and-plate viscometer [35–37]. The endothelial cells usually reached confluence under static culture and then were exposed to higher shear stresses. In this study, we tested different shear flow conditions within the variations of measured physiological range in venous system, including continues lower shear stresses (LSC-LST), continues higher shear stresses (HSC-HST), and changing shear stress from low to high (LSC-HST), respectively. Under higher flow conditions, the decreased cell surface area and increased cell density indicated a role of shear in promoting EC proliferation. This phenomena has also been resported by other studies [38]. The advantages of long term perfused microfluidic model also include the ability to gently remove the cells that were not fully attached during the cell seeding, and to continuously remove the wastes out of the channels and prevent over-confluence of the cell growth. We also noticed that within the microvessel channels, not all of the HUVECs aligned along the flow direction in each group. The Reynolds number (*Re*) for the selected microfluidic channel region was around 0.014 at the low flow rate (1 dyne/cm^2^) and 0.22 at the high flow rate (10 dyne/cm^2^) used during the media perfusion (Table 1). In fluid mechanics, Reynolds number is used to predict flow patterns. When Reynolds number is well below one, it indicates the fluid to be hydrodynamically stable with smooth and constant motion. Laminar flow occurs at low Reynolds numbers. Therefore, it was very unlikely existing of disturbed or secondary flow during the perfusion. Our results showed that the higher shear stress resulted in higher percentages of aligned cells. Based on the cell density counts, it is obvious that the cells within the channels have some variations of their growth stages, and therefore, showing different sensitivity to the shear stress or responding to the same shear stress at different rate. We would like to point out that, although effort and progress have been made to reduce the gaps between ECs *in vivo* and *in vitro*, current methods are still far from an ideal replication of all aspects of intact microvessels. One of the examples is that the proliferation rate of ECs in normal tissue is very low, estimated from months to years [39], but can be thousands times higher under stimulation or under cell culture conditions. The shear force, space restrictions, and the cell culture media composition may all affect the EC proliferation rate, and the variations of the cell growth stage within the microchannels may result in non-synchronized responses to mechanical stimuli.

The major technical improvement for biological applications reported in this study is the quantitative measurements of agonist-induced dynamic changes in endothelial [Ca^2+^]_i_ and NO production at individual cell levels in a well-developed microvessel network. Studies in individually perfused intact microvessels indicated that inflammatory mediator-induced increases in endothelial [Ca^2+^]_i_ is essential for increases in microvessel permeability and that the magnitude of the [Ca^2+^]_i_ determines the degree of permeability increases [15–18, 40]. In addition, agonist-induced Ca^2+^/calmodulin-dependent endothelial nitric oxide synthase (eNOS) activation and NO production have been shown to play important roles in the regulation of microvessel permeability [10–12]. In this study, we directly applied the methods developed in individually perfused intact microvessels [17, 26] to this cultured microvessel network and conducted parallel studies to those performed in the intact microvessels *in vivo*. Currently the uses of microfluidics to study intracellular calcium and NO responses have been reported by a few investigators. The calcium studies were either from suspended leukemic cells [41] or from non-confluent osteoblasts [42]. Though ATP-induced NO has been reported in bPAEC cultured microfluidic device, there was no characterization of the junctions of endothelial cells in which NO was measured and no temporal resolution of NO production was analyzed in those studies [23]. In our study, we choose ATP as the representative agonist to study receptor-mediated changes in endothelial [Ca^2+^]_i_ and NO production. ATP can be released by red blood cells, aggregated platelets, and injured tissue or under inflammatory conditions. The increased levels of ATP cause the release of endothelial-derived relaxing factor and trigger the synthesis of prostacyclin, and increase in microvessel permeability by increasing endothelial [Ca^2+^]_i_ [18, 43, 44]. The action of ATP is primarily via the purinergic P_2y_ receptor expressed on most types of endothelial cells [45, 46]. The calcium measurements using fluo-4 as indicator in the microvessel network showed similar responses to what we found in intact microvessels [11, 18]. The observed time courses of the changes in endothelial [Ca^2+^]_i_ were also similar to those found in intact microvessels [47]. The residence time for the ATP application in the microvessel model was 1.3 sec with the slowest flow rate (0.35μl/mins), and should not affect the measured responses.

As for NO measurements using DAF-2 DA, we need to recognize the specific manner of DAF-2 chemical conversion in the presence of NO and conduct data analysis accordingly [26, 48], which has not been presented in the previous reports. DAF-2 DA is membrane permeable and diffuses freely into the cells driven by concentration gradient and is then hydrolyzed by cytosolic esterase to form DAF-2. Intracellular DAF-2 is less membrane permeable due to its polarity, and is designed to be trapped inside the cells. Because the fluorescence chemical transformation of DAF-2 by NO is irreversible [48], the detected NO-sensitive fluorescence with DAF-2 represents a cumulative production of NO, not the dynamic changes of NO. The slope of the FI_DAF_ curve represents the NO production rate and the plateau indicates the termination of NO production instead of the actual NO concentration in the cells. To present the real-time course of NO production, df/d*t*, the NO production rate, was calculated based on the differential conversion of that regression equation (shown in Fig 7A). Using this method, our study presented the first measurements of the basal NO production rate and the changes in NO production after exposure to ATP in HUVECs-developed microvessels in the microfluidic device with temporal and spatial resolution. The results are comparable to those derived from individually perfused intact venules. Although venules have pericytes surrounding the vessel walls, currently no information suggests that pericytes influence agonist induced NO production in endothelial cells. When NO was measured in DAF-2 loaded microvessels, DAF-2 DA only loaded into ECs, not into pericytes, thus the DAF-2 fluorescence was only from ECs [11].

## Conclusions

These results presented in this paper demonstrated *in vitro* formation of a microvessel network that recapitulated key features of microvessels *in vivo* and validated its utility for biological applications. The fabrication process of the microchannels was simple and straightforward to lower the barrier for biologists. The cell seeding and culture with constant perfusion method was user friendly to most researchers and easy to replicate. Cells in our model kept their phenotype, viability, proliferation with proper barrier functions. Comparing to other conventional *in vitro* models, our model is more physiological realistic. In particular, HUVECs were successfully grown to confluence throughout entire microchannels under well-controlled conditions. The flow conditions applied to the cultured microvessels were close to that at the similar type of vasculature *in vivo*. Immunofluorescent staining of VE-cadherin demonstrated the formation of vascular endothelial junctions, an indication of well-maintained endothelial restrictive barrier functions. HUVECs responded to different physiologically relevant flow shear stresses and inflammatory stimuli. The endothelial [Ca^2+^]_i_, basal NO, and the changes in NO production rate in response to ATP were real-time measured and quantified at individual cellular level in Fluo 4 and DAF-2 loaded microvessels, respectively. Most importantly, the agonist-induced transient [Ca^2+^]_i_ responses and increased NO production were similar to those observed in individually perfused intact microvessels. The developed microvessel model has the potential to bridge the gap between over-simplified *in vitro* tests and more expensive and labour-intensive *in vivo* animal models for the microvascular signalling and functional studies. It offers valuable quantitative insights into how biophysical and biochemical properties influence vascular biology and pathophysiology, and serves as the complements for *in vivo* animal studies. Because the cultured endothelial cells can be from human tissues, a well characterized device will be suitable for studies of interactions between human blood components with microvessels formed by human endothelial cells under physiological and pathological conditions.
